# Investigation of Avian Influenza Infections in Wild Birds, Poultry and Humans in Eastern Dongting Lake, China

**DOI:** 10.1371/journal.pone.0095685

**Published:** 2014-04-22

**Authors:** Jinghong Shi, Lidong Gao, Yun Zhu, Tao Chen, Yunzhi Liu, Libo Dong, Fuqiang Liu, Hao Yang, Yahui Cai, Mingdong Yu, Yi Yao, Cuilin Xu, Xiangming Xiao, Yuelong Shu

**Affiliations:** 1 National Institute for Viral Disease Control and Prevention, Chinese Centre for Disease Control and Prevention, Key Laboratory for Medical Virology, National Health and Family Plan Commission, Beijing, China; 2 Hunan Provincial Center for Disease Control and Prevention, Changsha, China; 3 Yueyang City Centre for Disease Control and Prevention, Yueyang, China; 4 Hunan East Dongting Lake National Nature Reserve, Yueyang, China; 5 University of Oklahoma, Norman, Oklahoma, United States of America; Duke-NUS Gradute Medical School, Singapore

## Abstract

We investigated avian influenza infections in wild birds, poultry, and humans at Eastern Dongting Lake, China. We analyzed 6,621 environmental samples, including fresh fecal and water samples, from wild birds and domestic ducks that were collected from the Eastern Dongting Lake area from November 2011 to April 2012. We also conducted two cross-sectional serological studies in November 2011 and April 2012, with 1,050 serum samples collected from people exposed to wild birds and/or domestic ducks. Environmental samples were tested for the presence of avian influenza virus (AIV) using quantitative PCR assays and virus isolation techniques. Hemagglutination inhibition assays were used to detect antibodies against AIV H5N1, and microneutralization assays were used to confirm these results. Among the environmental samples from wild birds and domestic ducks, AIV prevalence was 5.19 and 5.32%, respectively. We isolated 39 and 5 AIVs from the fecal samples of wild birds and domestic ducks, respectively. Our analysis indicated 12 subtypes of AIV were present, suggesting that wild birds in the Eastern Dongting Lake area carried a diverse array of AIVs with low pathogenicity. We were unable to detect any antibodies against AIV H5N1 in humans, suggesting that human infection with H5N1 was rare in this region.

## Introduction

Human cases of highly pathogenic avian influenza (HPAI) H5N1 virus infection since the end of 2003 have been reported in 15 Eurasian countries. As of January 2014, there were 650 confirmed human cases of avian influenza A H5N1 infection reported, with a mortality rate exceeding 59% [1]. In China, 45 confirmed HPAI H5N1 cases were reported across 17 provinces and in Hong Kong, of which 30 died. The majority of cases were detected in winter and spring and most cases had a history of direct or indirect exposure to sick and/or dead birds [2]. Previous studies have shown that migratory birds play an important role in the geographical dissemination of avian influenza virus (AIV) H5N1 [3]. Migratory birds have also been considered to be the natural hosts of AIV. No clear conclusions have been made regarding the mode of transmission among wild birds, poultry, and humans [4].

We selected the Eastern Dongting Lake area in Hunan Province as our study site because seven confirmed human cases of AIV H5N1 have been reported in this area. Dongting Lake is located in the northeastern part of Hunan Province, and is centrally located along the Yangtze River. It is the second largest fresh water lake and the DongTing Lake wetland is one of the largest lacustrine wetland eco-system in China [5].The Eastern Dongting Lake area is the largest, accounting for half of the Dongting Lake. Dongting Lake is also a major stopover destination and overwintering area for birds along the East Asia-Australia migratory flyway, especially Eastern Dongting Lake as a typical subtropical inland wetlands. During winter, more than 10 million birds of nearly 300 species congregate in Eastern Dongting Lake [6]. The human population around Dongting Lake is 15 million, accounting for 23.8% of the total population in Hunan Province [7]. More than 90% of local residents depend on Dongting Lake and its wetlands [8]. Duck farming is prevalent in the lake area, with more than 80 million birds raised in this area in 2007 [9].

In this study, we sought to determine the prevalence of AIVs in wild birds and domestic ducks, and evaluate the risk of human infection with H5N1 among people exposed to wild birds and/or domestic ducks in the Eastern Dongting Lake area.

## Materials and Methods

### Ethics Approval

Our study was conducted following approval by the Chinese Center for Disease Control and Prevention Ethical Review Committee (201118). All participants provided signed informed consent forms.

### Environmental Sample Collection

Wild bird sample collection sites were designated by the Forestry Administration and Hunan East Dongting Lake Nature Reserve. Fresh fecal samples from wild birds and water samples were collected every month from November 2011 to April 2012. We collected one to four fresh fecal samples at each sampling point in the flood plain. Water samples were collected from locations where a clear footprint attributable to a waterfowl was seen. There was at least 10 m between two sampling points.

We collected fresh fecal and water samples from duck farms surrounding the Eastern Dongting lake area. These farms were within about a 5 km straight-line distance to the lake area. Inclusion criteria for duck farms included: ducks were being raised during our field survey; and there was a possible shared water source between domestic ducks and wild birds. We referred to fecal and water samples collectively as environmental samples.

### Serum Sample Collection

Two cross-sectional serological surveys were conducted in November 2011 and April 2012. Staff of the Eastern Dongting Lake Wetland Nature Reserve, local staff on duck farms, and individual duck breeders residing along the Eastern Dongting Lake were enrolled. Venous blood was collected by registered nurses. Inclusion criteria of participants were: they were local residents or individuals that had been in the area longer than 2 weeks; they signed the informed consent form; they were aged 18 years or older; and they had been exposed to wild birds and/or domestic ducks.

### Sample Storage and Transportation

Fecal samples were placed in tubes containing M199 solution [0.5% (w/v) bovine serum albumin (BSA), 2×106 U/L penicillin, 200 mg/L streptomycin, 2×106 U/L polymyxin B, 250 mg/L gentamycin, 60 mg/L levofloxacin hydrochloride, and 5×105 U/L nystatin] [32]. Water samples were placed in empty tubes. Serum and environmental samples were transported to the influenza surveillance laboratory of the local center for disease control (CDC) at 4°C within 24 h of collection. Serum and environmental samples were stored at −20 and −70°C, respectively. Samples were transported in accordance with national biosafety regulations; all samples were tested by the Chinese National Influenza Center.

### Nucleic Acid Testing, Virus Isolation, and Subtype Identification

Total RNA was extracted and purified from sample aliquots (200 µL) using a QIAamp One-For-All Nucleic Acid Kit (Qiagen, Germany) and the BioRobot Universal System (Qiagen), following the manufacturer’s protocol. The RNA of AIVs was detected using quantitative polymerase chain reaction (qPCR) assays (AgPath, Foster City, CA, USA) targeting the Matrix gene. Samples that were positive were inoculated in the allantoic cavity of 9-day-old embryonated chicken eggs (ECEs). The ECEs were incubated at 35°C for 48 h, then chilled at 4°C overnight. Allantoic fluid was harvested and hemagglutination assays were performed with 1% turkey red blood cells to confirm the presence of viruses. Viral RNA was extracted using an RNeasy Mini Kit (Qiagen) following the manufacturer’s instructions. RNA was reverse transcribed into cDNA with a SuperRT cDNA Kit (CWBIO, Beijing, China) using the Uni12 primer (5′-AGC AAA AGC AGG-3′). Identification of isolate subtypes was performed by PCR using 16 sets of primers specific for hemagglutinin (HA; H1–H16) and nine sets specific for neuraminidase (NA; N1–N9) that were designed by the Chinese National Influenza Center.

### Sequencing Analysis

Extracted viral RNA was reverse transcribed into cDNA using SuperRT cDNA Kit (CWBIO) and the aforementioned Uni12 primer. Reactions were incubated at 25°C for 10 min and then 42°C for 80 min. A 2×Es Taq MasterMix Kit (CWBIO) was used to amplify virus genes with the aid of eight gene segment-specific primers. Amplified products were purified using a QIAquick PCR purification kit (Qiagen). Sequencing reactions were carried out with a BigDye Terminator v3.1 Cycle sequencing kit (AB, Foster City, CA, USA) on an ABI PRISM 3700xl DNA sequencer following the manufacturer’s instructions.

### Phylogenetic Analysis

Lasergene 8.1 software (DNASTAR) was used to assemble gene sequences and Mafft 6 was used for the comparison of sequences. The neighbor joining (NJ) algorithm with a bootstrap value of 1,000 was used within MEGA 4.0 software. Phylogenetic trees were created for eight genes of AIV H5N1. All gene sequences for comparison were acquired from the GenBank influenza database.

### Serological Screening Assays

We conducted hemagglutination-inhibition (HI) assays using horse red blood cells for preliminary screening of HPAI H5N1-specific antibodies. Microneutralization (MN) assays were used to confirm results when an HI titer of 20 or greater was obtained [31]. We used two H5N1 viruses [A/Anhui/1/2005 (H5N1) virus (clade 2.3.4) and A/domestic duck/Yueyang/C0816/2012 (H5N1) virus (clade 2.3.2.1)] as antigens in this study for HI and MN assays.

### Statistical Analysis

We initially entered our data into a customized database (Epidata3.0) and then transferred it to SPSS (SPSS Inc., Chicago, IL, USA) for statistical analysis. Differences between proportions were analyzed using the χ2 test. A P-value less than 0.05 was considered significant.

## Results

### Detection of AIVs

**Figure 1:**
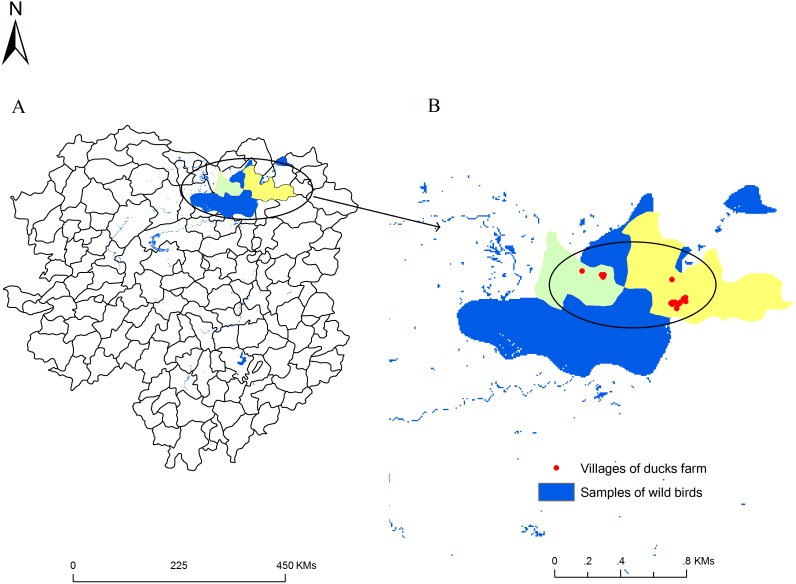
Sample collection sites around the Eastern Dongting Lake area. (A) Hunan Province. Dongting Lake is located to the north of Hunan Province (black circle). (B) Dongting Lake. The black circles indicate sample collection areas. Junshan district (green). Yueyang county (yellow) surrounding the Eastern Dongting Lake area. Villages where environmental samples from duck farms are shown in red.

**Figure 2:**
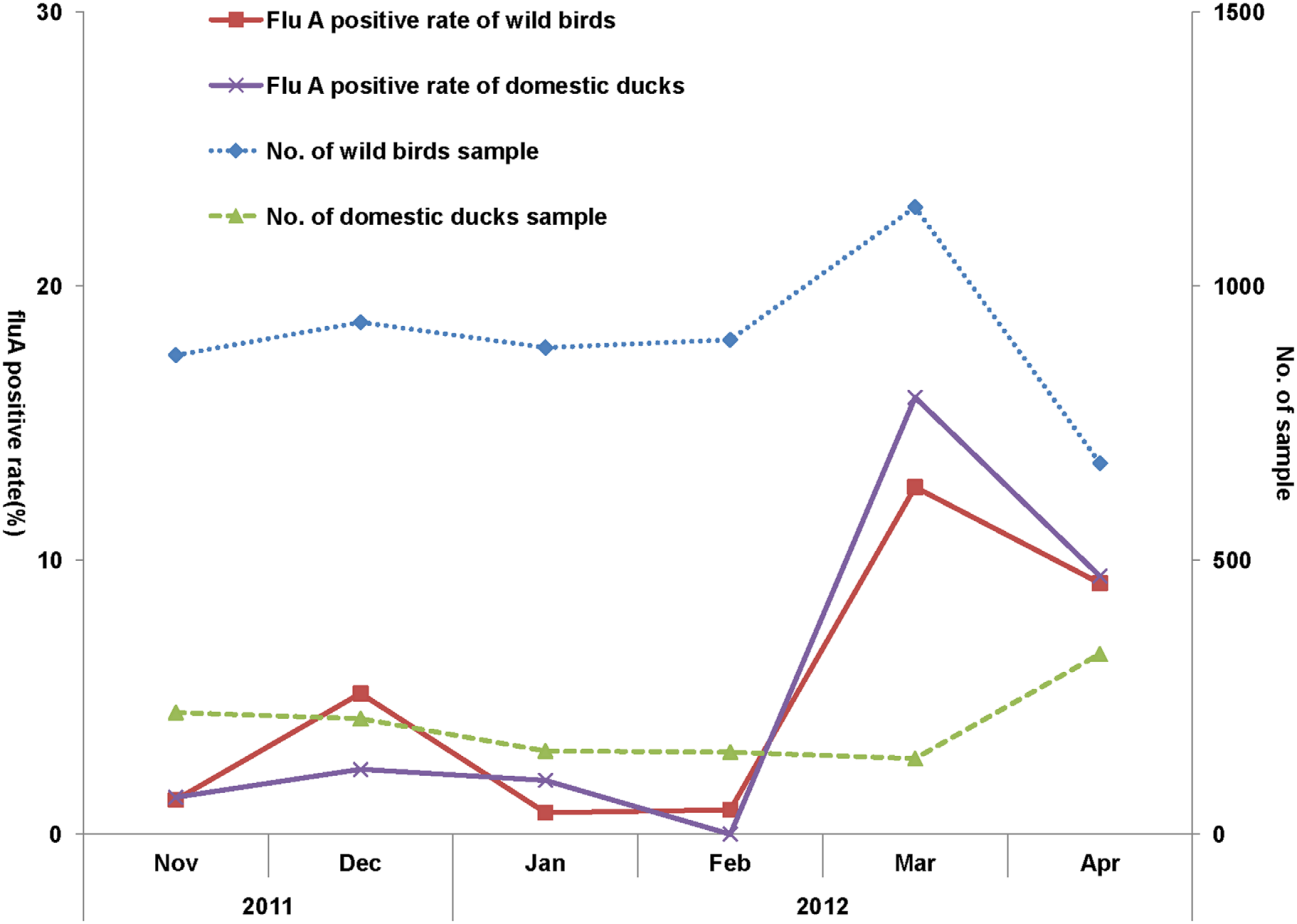
Time distribution of AIV prevalence in wild birds and domestic ducks.

We collected 6,621 environmental samples from November 2011 to April 2012, of which 5,419 were from wild birds and 1,202 from domestic ducks (Table 1 and Figure 1). We detected AIVs in 5.19 and 5.32% of samples from wild birds and domestic ducks, respectively (χ2 = 0.0384, P = 0.84). We observed two peaks for virus prevalence among wild birds, in December 2011 and March 2012. A peak in prevalence was seen for domestic ducks in March 2012 (Figure 2). There was a greater proportion of AIV-positive fecal samples than water samples for wild birds (χ2 = 30.4556, P<0.001), however this was not significantly different for ducks (χ2 = 0.3841, P = 0.54). There was a higher proportion of AIV-positives among the water samples associated with ducks than those with wild birds (χ2 = 15.9069, P<0.001); the same trend was not seen with fecal samples (χ2 = 1.1357, P = 0.29) (Table 2). The prevalence of AIV positives varied among the different wild bird species, with the greatest proportion seen in Anatidae birds (5.15%), followed by Charadriiformes (0.44%) and then members of the Ardeidae family (0.18%) (Table 3).

**Table 1:** Summary of samples analyzed in this study.

		Wild birds			Domestic Ducks			
Year	month	Feces	Water	Total	Feces	Water	Total	*Total*
2011	11	713	161	874	134	88	222	1096
	12	772	162	934	136	75	211	1145
2012	1	706	182	888	92	60	152	1040
	2	757	145	902	90	60	150	1052
	3	911	233	1144	82	56	138	1282
	4	506	171	677	205	124	329	1006
*Total*		4365	1054	5419	739	463	1202	6621

**Table 2:** Prevalence of AIV in samples collected from wild birds and domestic ducks.

Type of specimen	wild birds(%)	domestic ducks(%)	P value
feces	6(262/4365)	5.01(37/739)	0.29
water	1.8(19/1054)	5.83(27/463)	<0.001
Total	5.19(281/5419)*	5.32(64/1202)**	0.84

*P<0.001 between fecal and water samples among wild birds.

**P = 0.54 between fecal and water samples among domestic ducks.

**Table 3:** Detection of AIVs in samples collected from wild birds and domestic ducks.

Species	FluA positiverate(%)	Flu A positivestandard rate(%)	virus isolates
Domestic ducks	5.32(64/1202)		H5N1(1),H4N2(2),H3N6(2)
Wild birds	5.19(281/5419)		
Anatidae	7.12(225/3160)	5.15	H1N2(1),H1N5(5),H1N8(2),H6N1(1),H6N2(2),H7N7(1),H9N2(20)
Charadriiformes	21.11 (19/90)	0.44	H9N2(2)
Ardeidae	1.73(8/463)	0.18	H5N2(2)
Unknown	1.53(10/652)	0.23	H12N8(1),H6N1(1),H5N2(1)

### Virus Isolation

**Figure 3:**
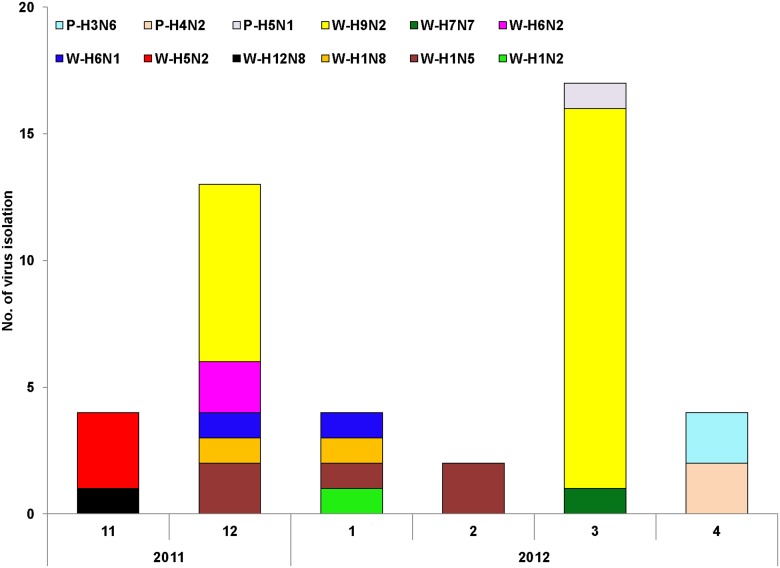
Monthly distribution for AIV isolation. ‘P’ indicates virus isolated from the feces of domestic ducks. ‘W’ indicates virus isolated from the feces of wild birds.

We isolated 39 AIV strains from fecal specimens of wild birds (Table 3), including 22 H9N2, 5 H1N5, 3 H5N2, 2 H1N8, 2 H6N1, 2 H6N2, 1 H1N2, 1 H7N7, and 1 H12N8; of these, 32 strains were isolated from Anatidae birds, 2 from Ardeidae birds, 2 from Charadriiformes, and 3 from unidentified species. We isolated five avian influenza virus strains from fecal samples of domestic ducks (Table 3), including 1 H5N1, 2 H4N2, and 2 H3N6 strains. No viruses were isolated from water samples. There was no statistical differences in the proportion of virus strains isolated from wild birds and domestic ducks (0.72% and 0.42%, respectively). Among different species of wild birds, virus isolation rates differed among the Anatidae (0.73%), Charadriiformes (0.05%), and the Ardeidae (0.05%). The number of AIVs was more in December and March. The trends in numbers of virus strains isolated were similar to those for the prevalence of AIV in samples. (Figure 3).

### Molecular Characterization of an HPAI H5N1 Virus

The A/Duck/Yueyang/C0816/2012 (H5N1) strain was isolated from the feces of a domestic duck. Phylogenetic analysis showed that all eight genes were most closely related to clade 2.3.2.1 H5N1 viruses [A/Hubei/1/2010 (H5N1)-like] currently circulating in China (Figure S1, Figure S2). The cleavage site of the HA protein for A/Duck/Yueyang/C0816/2012 contained multiple basic amino acids (PQIERRRRKR↓GL). The HA receptor binding sites contained 226Q and 228G (H3 numbering), indicating preferential binding for α-2,3 sialic acid. A 20-amino acid deletion (amino acids 49–68) was observed in the NA protein, and the virus was sensitive to NA inhibitors based on the sequence of the NA protein (275H).

### Serological Survey

We collected 1,050 human serum samples from 515 men and 535 women; approximately 82% of subjects were older than 40 years. Of the subjects, 981 had been exposed to healthy poultry, 55 to sick and/or dead poultry, 76 to healthy wild birds, and 22 to sick and/or dead wild birds. When we used A/Anhui/1/2005 (H5N1) as the detecting antigen, the H5N1 antibody titer in 106 samples was greater than or equal to 20. When we used A/Duck/Yueyang/C0816/2012 (H5N1) as the detecting antigen, the H5N1 antibody titer was greater than 10 in 76 samples with the highest titer being 80. The female participant (1∶80) was a 63-year-old involved in the fishing industry who had lived on a boat in the lake area for 17 years (Table 4). However, none of these positive results were confirmed by MN assays.

**Table 4:** Serological survey of human serum samples for the presence of antibodies against AIV H5N1.

			A/Duck/Yueyang/C0816/2012 (H5N1)					A/Anhui/1/2005 (H5N1)			
Category	Subcategory	n	1∶20	1∶40	1∶80	≥1∶20	<1∶20	1∶20	1∶40	≥1∶20	<1∶20
Age (years)	≥60	276	15	14	1	30	246	30	3	33	243
	40–59	590	27	13	0	40	550	48	0	48	542
	18–39	182	4	2	0	6	176	25	0	25	157
	unknown	2	0	0	0	0	2	0	0	0	2
Gender	Male	515	26	18	0	44	471	60	2	62	453
	Female	535	20	11	1	32	503	43	1	44	491
healthy poultry	yes	981	44	27	1	72	909	95	3	98	883
	no	69	2	2	0	4	65	8	0	8	61
sick/dead poultry	yes	55	1	1	0	2	53	4		4	51
	no	990	45	28	1	74	916	99	3	102	888
	unknown	5	0	0	0	0	5	0	0	0	5
healthy wild birds	yes	76	4	2	0	6	70	16	0	16	60
	no	974	42	27	1	70	904	87	3	90	884
sick/dead wild birds	yes	22	2	1	0	3	19	4	0	4	18
	no	1026	44	28	1	73	953	99	3	102	924
	unknown	2	0	0	0	0	2	0	0	0	2

## Discussion

Waterfowl, especially Anseriformes and Charadriiformes species, are considered to be the natural reservoir of influenza A virus, while domesticated ducks are important intermediate hosts in the spread of AIVs [10]. The Dongting Lake wetland serves as a natural seasonal shelter for a large number of migratory birds over winter and spring, therefore it is a suitable area to study AIV transmission among humans, domesticated ducks, and wild birds.

Several studies have shown that the prevalence of AIVs in wild birds is 2.6–3.6% in the wetlands serving as overwintering sites for wild birds [11]–[13]. Our findings show that AIV prevalence in environmental samples associated with wild birds was 5.19%. Prevalence peaked twice, in December and March, along with migration levels. The number of wild birds started to increase in November, peaking between December to February [14], [15]. The December peak in AIV prevalence could be related to the arrival of wild birds to the Dongting Lake area over winter. The peak in March was likely related to the stopover of wild birds during their migration from the south to north [15]. Some bird species might be more vulnerable to AIV infection; further study in this geographical region is required.

We found that AIV prevalence was similar between wild birds and domestic ducks, with a similar distribution. This is possibly because of wild birds and domestic ducks living in a common environment. In this study, only duck farms with water sources, such as a pond or a ditch, around the Eastern Dongting Lake were selected. High-density contact between wild birds and domestic ducks could contribute to the increased prevalence of AIV. However, there is no direct evidence for the transmission of AIVs from wild birds to ducks, or vice versa.

Previous studies have shown that H5N1, H9N2, and H10N8 AIVs have been isolated from wild birds in the Dongting Lake wetlands [16]–[19]. H1N5, H1N2, H1N8, H5N2, H6N1, H6N2, H7N7, H9N2, and H12N8 AIVs were isolated from wild birds in this study, indicating increased diversity of low pathogenicity AIVs circulating in wild birds. It is possible these birds are the reservoir host of AIVs in this area [20]. The rate of virus isolation from wild bird samples was 0.72%, similar to that for migratory duck samples (0.78%) as determined by Guan et al [21]. Viruses were mainly isolated from Anatidae and Charadriiformes birds with a higher prevalence of AIV, consistent with results reported by Alexander [22]. These findings highlight the importance of being more vigilant with regard to AIV surveillance in important migratory bird species.

In our study, two H4N2, two H3N6, and one H5N1 strain were isolated from the feces of domestic ducks. All strains, except for H5N1, were considered AIVs with low pathogenicity. All eight genes of the HPAI H5N1 virus were similar to other clade 2.3.2.1 viruses currently circulating in China, suggesting that the H5N1 virus we isolated was not transmitted from wild birds.

All the AIVs we isolated from wild birds and domestic ducks were from fecal samples. We were unable to isolate viruses from any of the water samples, possibly because AIVs were able to survive for longer periods in fecal samples [23], [24]. We proposed that it is better to collect fecal specimens for AIV surveillance. Our data do not show that the H5N1 virus circulating in poultry was not from wild birds because of the limited numbers of samples we examined. However, we did show that the profiles of AIVs circulating in wild birds and ducks in that area were obviously different.

Among people exposed to birds, the prevalence of H5N1 antibodies is in the range of 0–14.6% [25], [26]. We were unable to detect any H5N1 antibodies in human serum samples using an MN assay, indicating that the risk of HPAI infection was very low despite contact with wild birds and/or domestic ducks. The results from our study were consistent with those from other studies [27]–[30].

In conclusion, we showed that wild birds and ducks around the Eastern Dongting Lake area carried a diverse range of low pathogenicity AIVs. Several AIV subtypes were detected for the first time, and a HPAI H5N1 virus was detected in domestic ducks. Our findings highlight the importance of maintaining AIV surveillance, and early detection of reassortment between HPAI H5N1 viruses and low pathogenicity AIVs.

## Supporting Information

Figure S1 **Phylogenetic trees based on the HA genes of the strain isolated from a domestic duck in Yueyang, Hunan Province, China.** Phylogenetic trees were generated using the neighbor joining method. Reliability of the tree was assessed by bootstrap analysis with 1000 replicates. Values above branches indicate neighbor joining bootstrap values. Isolates from our current study are marked in red.
(PNG)

Figure S2 **Phylogenetic trees based on the NA genes of the strain isolated from a domestic duck in Yueyang, Hunan Province, China.** Phylogenetic trees were generated using the neighbor joining method. Reliability of the tree was assessed by bootstrap analysis with 1000 replicates. Values above branches indicate neighbor joining bootstrap values. Isolates from our current study are marked in red.
(PNG)
