# Supplementary figures and images for: Investigation of Avian Influenza Infections in Wild Birds, Poultry and Humans in Eastern Dongting Lake, China
Source: PLoS One. 2014 Apr 22;9(4):e95685. doi: 10.1371/journal.pone.0095685 (PMC3995770; doi:10.1371/journal.pone.0095685)

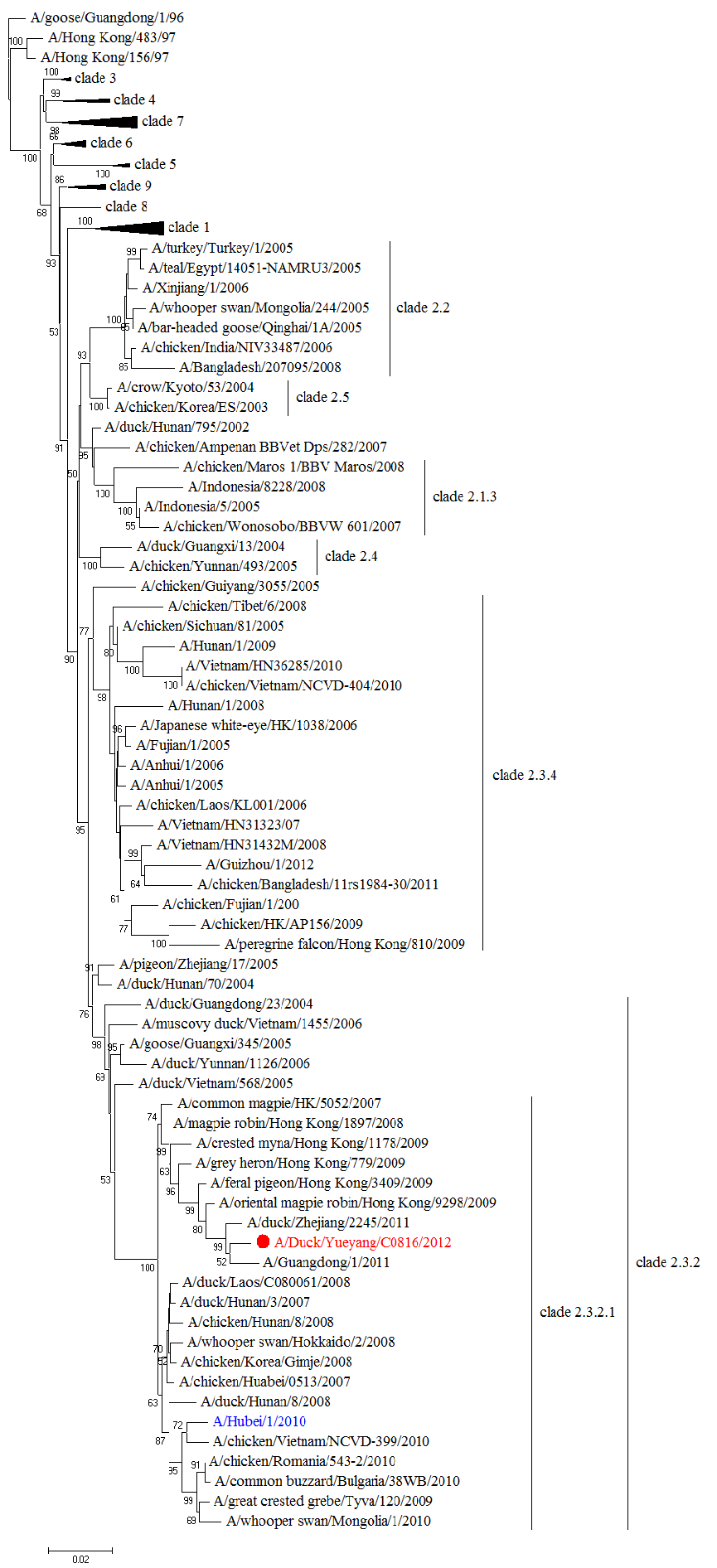

Supplement: Figure S1 — Phylogenetic trees based on the HA genes of the strain isolated from a domestic duck in Yueyang, Hunan Province, China. Phylogenetic trees were generated using the neighbor joining method. Reliability of the tree was assessed by bootstrap analysis with 1000 replicates. Values above branches indicate neighbor joining bootstrap values. Isolates from our current study are marked in red. (PNG) [file pone.0095685.s001.png]

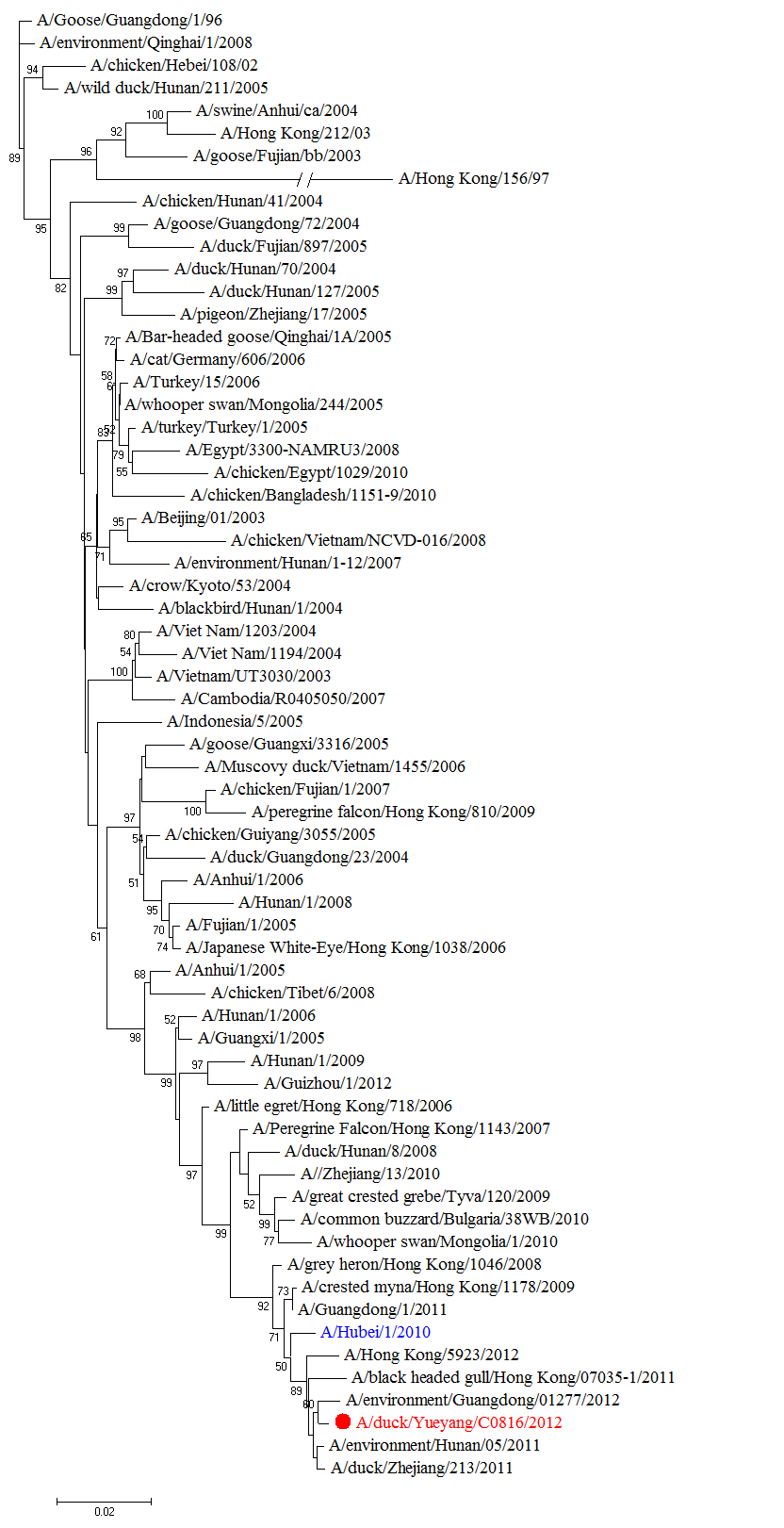

Supplement: Figure S2 — Phylogenetic trees based on the NA genes of the strain isolated from a domestic duck in Yueyang, Hunan Province, China. Phylogenetic trees were generated using the neighbor joining method. Reliability of the tree was assessed by bootstrap analysis with 1000 replicates. Values above branches indicate neighbor joining bootstrap values. Isolates from our current study are marked in red. (PNG) [file pone.0095685.s002.png]
